# Transcription factor c-Jun modulates GLUT1 in glycolysis and breast cancer metastasis

**DOI:** 10.1186/s12885-022-10393-x

**Published:** 2022-12-07

**Authors:** Ping Zhu, Guoping Liu, Xue Wang, Jingjing Lu, Yue Zhou, Shuyi Chen, Yabiao Gao, Chaofu Wang, Jerry Yu, Yangbai Sun, Ping Zhou

**Affiliations:** 1 Department of Pathology and Musculoskeletal Oncology of Shanghai Cancer Center; Department of Physiology and Pathophysiology of School of Basic Medical Sciences, Fudan University, No. 270, 130 Dongan Road, Shanghai, 200032 China; 2 Department of General Surgery, Xin Hua Hospital Affiliated to Shanghai Jiao Tong University School of Medicine, Shanghai, 200092 People’s Republic of China; 3 Department of Pathology, Ruijin Hospital, Shanghai Jiaotong University School of Medicine, Shanghai, 200025 China; 4 Department of Medicine, University of Louisville, Louisville, KY 40292 USA

**Keywords:** Breast neoplasms, Neoplasm metastasis, GLUT1, Glycolysis, C-Jun

## Abstract

As the main isoforms of membranous glucose transporters (GLUT), GLUT1 involves tumorigenesis, metastasis and prognosis in a variety of cancers. However, its role in breast cancer metastasis remains to be elucidated. Here we examined its transcriptional and survival data in patients with breast cancer from several independent databases including the Oncomine, Gene Expression Profiling Interactive Analysis, Gene Expression across Normal and Tumor tissue, UALCAN, cBioPortal, Kaplan-Meier Plotter and PROGgeneV2. We found that its mRNA expression was significantly high in cancer tissues, which was associated with metastasis and poor survival. Transcription factor c-Jun might bind to GLUT1 promoter to downregulate its gene expression or mRNA stability, therefore to suppress glycolysis and metastasis. By qRT-PCR, we verified that GLUT1 was significantly increased in 38 paired human breast cancer samples while JUN was decreased. Furthermore, the protein level of GLUT1 was higher in tumor than in normal tissues by IHC assay. To explore underlying pathways, we further performed GO and KEGG analysis of genes related to GLUT1 and JUN and found that GLUT1 was increased by transcription factor c-Jun in breast cancer tissues to influence glycolysis and breast cancer metastasis.

**Supplementary Information:**

The online version contains supplementary material available at 10.1186/s12885-022-10393-x.

## Background

Breast cancer is the most common cancer with the highest mortality in females worldwide. Approximately 2.1 million women were diagnosed with breast cancer in 2018, accounting for almost 25% of cancer cases in females [1]. Metastasis is the leading cause of cancer-related death (about 90%) [2, 3]. Prognosis remains disappointing in spite of advances in surgery, radiotherapy, chemotherapy, immunotherapy and targeted therapy. Thus, it is important to understand the molecular mechanisms underlying the metastatic process.

Altered metabolism increases cell survival and/or proliferation as well as the metastatic potential in various cancers, including the breast cancer. Warburg effect, also known as aerobic glycolysis (though it occurs also under anaerobic conditions in cancers), is a well-known dysregulation of cancer metabolism with increased glucose consumption and lactic acid production [4]. Dysregulated glucose metabolism is a hallmark in human cancer [5–7]. Glucose is the primary energy source for mammalian cells. Cancer cells typically rely on aerobic glycolysis to meet their biosynthetic need for energy, nucleotides, lipids, and amino acids, thus promoting cell survival and proliferation [6]. However, rapidly proliferating cancer cells can maximally elevate glucose uptake and therefore dramatically increases expression of membranous glucose transporters (GLUT). There are four main isoforms (GLUT1, GLUT2, GLUT3, GLUT4). GLUT1 overexpresses in most cancers and associates with poor survival [8, 9]. As a primary switch of aerobic glycolysis, GLUT1 is also known as the solute carrier family 2 member 1 protein SLC2A1, involving in tumor survival and metastasis [7, 10–12]. GLUT1 accelerates aerobic glycolysis and provides energy and biomass for the movement of primary tumor cells to distant locations. However, its regulatory mechanism in breast cancer metastasis remains unclear.

In recent years, many mutated or dysregulated transcription factor targets were found in cancers. They are a distinct class of anticancer drug targets that modulate aberrant gene expression, including genes related to carbohydrate metabolism and metastasis [13–15]. c-Jun encoded by gene JUN is one of the important cancer-related transcription factors and is a key molecule in glucose metabolism and cancer metastasis. However, its relation to GLUT1 in glucose metabolism-mediated metastasis in breast cancer has not been fully investigated. The potential mechanism of GLUT1 in glucose metabolism pathway on the occurrence and development of breast cancer needs to be further clarified. We suspect that the interaction between GLUT1 and c-Jun may be an important cause why GLUT1 affects the progression of breast cancer through metabolic reprogramming. In this study, we used bioinformatics to comprehensively analyze their expression patterns and their relationship with survival, and verified by morphological experiments to clarify the regulatory role of GLUT1-c-Jun axis in breast cancer metastasis, and provide a new strategy for the treatment of breast cancer.

## Methods

### Clinical samples

Thirty-eight paired breast cancer and normal adjacent tissue samples were collected from patients, undergoing surgery in 2018 in the Department of Breast Surgery, Zhongshan Hospital, Fudan University, Shanghai, China. Each sample was divided into two parts. One part was frozen in liquid nitrogen within 60 min and then stored at − 80 °C until analysis. The remaining part was formalin fixed and paraffin-embedded for immunohistochemistry analysis. This study was approved by the Medical Ethics Committee of Fudan University.

### Quantitative reverse transcription-polymerase chain reaction analysis

Total RNA was isolated from clinical tissues with Trizol reagent (Invitrogen); reverse transcription reactions with ReverTra Ace qPCR RT Kit (TOYOBO), and qRT-PCR with HieffTMqPCR SYBR& Green Master Mix on ABI 7300 Fast Real-Time PCR System (Applied Biosystems), according to the manufacturer’s instructions [16]. The primer sequences of GAPDH are GGACCAATACGACCAAATCCG (forward) and AGCCACATCGCTCAGACAC (reverse). The primer sequences of GLUT1 are GGCCAAGAGTGTGCTAAAGAA (forward) and ACAGCGTTGATGCCAGACAG (reverse). The primer sequences of c-Jun are AACAGGTGGCACAGCTTAAAC (forward) and CAACTGCTGCGTTAGCATGAG (reverse). Relative portions of mRNAs were normalized to β-actin. Fold-change was calculated with standard ΔΔCt method.

### Immunohistochemistry

Paraffin-embedded tissue samples were deparaffinized at 65 °C for 40 min, cleared in xylene, rehydrated in a graded series of alcohol, and then heated with saline sodium citrate buffer at 95° to 100 °C for antigen retrieval. After cooling and two PBS washes, slides were incubated with primary antibody (GLUT1, PA6583, Elabscience) diluted in blocking solution at 4 °C overnight, followed by a horseradish peroxidase (HRP)-conjugated secondary antibody incubation at room temperature for 1 h. Slides were then developed with 3,3′-diaminobenzidine (DAB), counterstained with hematoxylin, and dehydrated in graded ethanols. Positive staining density was detected under a light microscope [16].

### Oncomine analysis

The transcription level of GLUT1 in various types of cancers was analyzed with Oncomine database [17] (https://www.oncomine.org/resource/login.html-The website has been shut down from 17.Jan 2022). This data will be available from the corresponding author on reasonable request.

### GEPIA database

GEPIA (http://gepia.cancer-pku.cn/index.html) is a newly developed interactive web server for analyzing RNA sequencing expression data [18].

### GENT database

Tisssue-wide gene expression patterns across 72 paired tissues and significant test results by two-sample T test are examined with GENT database (Gene Expression across Normal and Tumor tissue) [19] (http://gent2.appex.kr/gent2/).

### UALCAN database

UALCAN (http://ualcan.path.uab.edu/) is a comprehensive, friendly, and mutual web for inspecting cancer OMICS data [20].

### cBioPortal web tool

The breast invasive carcinoma (The Cancer Genome Atlas, Firehose Legacy) dataset was further analyzed by cBioPortal [21] (https://www.cbioportal.org/). The genomic profiles of GLUT1 include mutations, putative copy number alterations from genomic identification of significant targets in cancer (GISTIC) and mRNA expression Z scores (microarray).

### The human protein atlas

The Human Protein Atlas (https://www.proteinatlas.org/) is a Swedish-based program initiated in 2003. It maps all the human proteins in the cell, tissue and organ with integration of various omics technologies, including antibody-based imaging, mass spectrometry-based proteomics, transcriptomics and systems biology [22].

### Kaplan-Meier plotter

Kaplan-Meier Plotter contains gene expression and survival data of breast cancer patients (http://kmplot.com/analysis/index.php?p=background) [23]. The best probe set (201249_at) of GLUT1 was used to obtain the plots. Patient samples were splited into two groups by auto selection of best cutoff value.

### PROGgeneV2 database

PROGgeneV2 database (http://www.progtools.net/gene/)is for survival analysis on single genes, or on mean expression of a group of user defined genes including 27 types of tissue [24].

### MethSurv web tool

MethSurv web tool (https://biit.cs.ut.ee/methsurv) was used to analyze the relationship between gene methylation and prognosis [25]. This data will be available from the corresponding author on reasonable request.

### Bc-GenExMiner web tool

Breast Cancer Gene-Expression Miner (bc-GenExMiner) is a new mining module that analyzes breast cancer gene-expression correlation and gene prognosis (http://bcgenex.ico.unicancer.fr) [26].

### GO and KEGG analysis

Gene ontology (GO) and Kyoto Encyclopedia of Genes and Genomes (KEGG) analysis were explored by R/Bioconductor with a cluster Profiler package.

## Results

### GLUT1 transcription level increased in Most types of Cancer

**Fig. 1:**
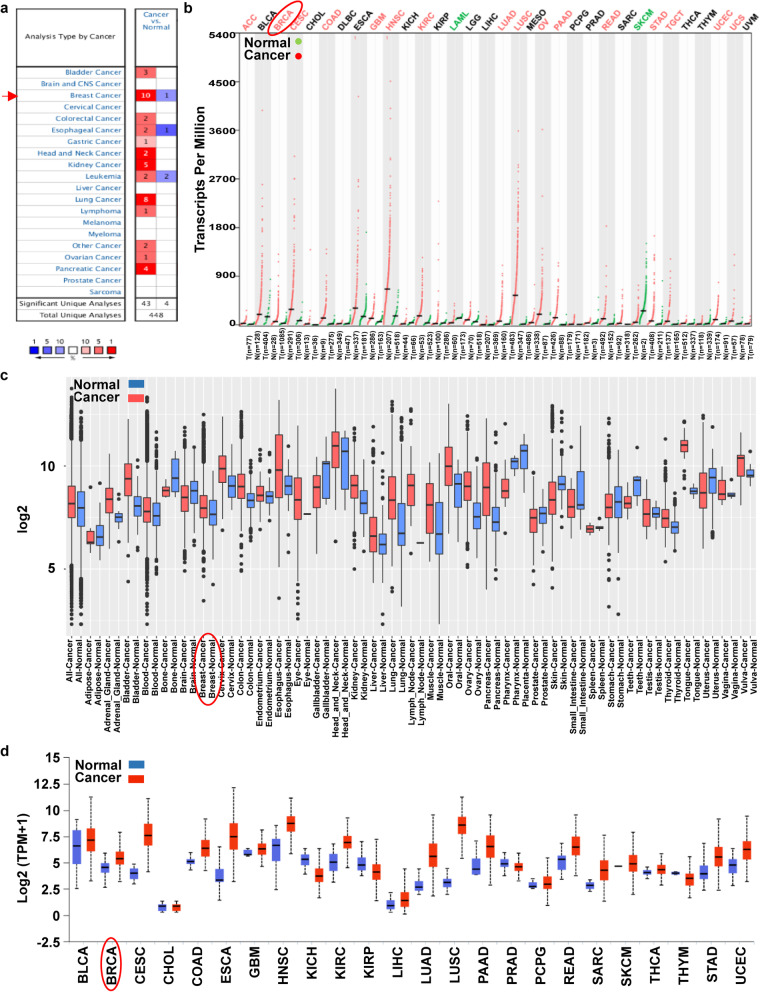
Transcription Level of GLUT1 in Different Kinds of Cancer versus their Corresponding Normal Samples. **a** Number of datasets contains GLUT1 mRNA expression (red: over expression, blue: low expression) from Oncomine database. Criterions: *p*-value: 0.0001, Fold change: 2, the rank of gene: 10%. The best gene rank percentile of the analyses determined the chamber color. **b** The expression level of the mRNA from the TCGA datasets in GEPIA database. Each dot represents one sample. **c** Box plot represent the expression pattern in GPL570 Platform (HG-U133_Plus_2) from the GENT database. Box represents the median, and 25th and 75th percentiles. And the dots symbolize outliers. **d** The GLUT1 mRNA expression in TCGA datasets from UALCAN database. Box represents the median, and 25th and 75th percentiles. (ACC: Adrenocortical carcinoma, BLCA: Bladder Urothelial Carcinoma, BRCA: Breast invasive carcinoma, CESC: Cervical squamous cell carcinoma and endocervical adenocarcinoma, CHOL: Cholangio carcinoma, COAD: Colon adenocarcinoma, DLBC: Lymphoid Neoplasm Diffuse Large B-cell Lymphoma, ESCA: Esophageal carcinoma, GBM: Glioblastoma multiforme, HNSC: Head and Neck squamous cell carcinoma, KICH: Kidney Chromophobe, KIRC: Kidney renal clear cell carcinoma, KIRP: Kidney renal papillary cell carcinoma, LAML: Acute Myeloid Leukemia, LGG: Brain Lower Grade Glioma, LIHC: Liver hepatocellular carcinoma, LUAD: Lung adenocarcinoma, LUSC: Lung squamous cell carcinoma, MESO: Mesothelioma, OV: Ovarian serous cystadenocarcinoma, PAAD: Pancreatic adenocarcinoma, PCPG: Pheochromocytoma and Paraganglioma, PRAD: Prostate adenocarcinoma, READ: Rectum adenocarcinoma, SARC: Sarcoma, SKCM: Skin Cutaneous Melanoma, STAD: Stomach adenocarcinoma, TGCT: Testicular Germ Cell Tumors, THCA: Thyroid carcinoma, THYM: Thymoma, UCEC: Uterine Corpus Endometrial Carcinoma, UCS: Uterine Carcinosarcoma, UVM: Uveal Melanoma)

We assessed GLUT1 mRNA expression in cancer and its corresponding normal tissue with four independent cancer data mining databases. 1) Oncomine database. Upregulation was found in almost all cancers, including breast, lung, kidney, pancreatic, bladder, head and neck, colorectal, esophageal, gastric, ovarian cancer, leukemia and lymphoma (Fig. 1a) with the greatest in breast cancer (10 up and 1 down datasets). 2) A combined TCGA and GTEx database. We examined the mRNA expression in 33 types of human cancer (Fig. 1b). The expression was significantly high in 16 types and low in 2 types. 3) The mRNA expression of GLUT1 at GPL570 Platform (HG-U133_Plus_2) in the GENT database. We found GLUT1 overexpressed in certain cancers, including breast cancer (Fig. 1c). 4) UALCAN web tool. The expression was significantly high in most cancers, including the breast (Fig. 1d).

### GLUT1 mRNA and protein increased in breast Cancer

**Fig. 2:**
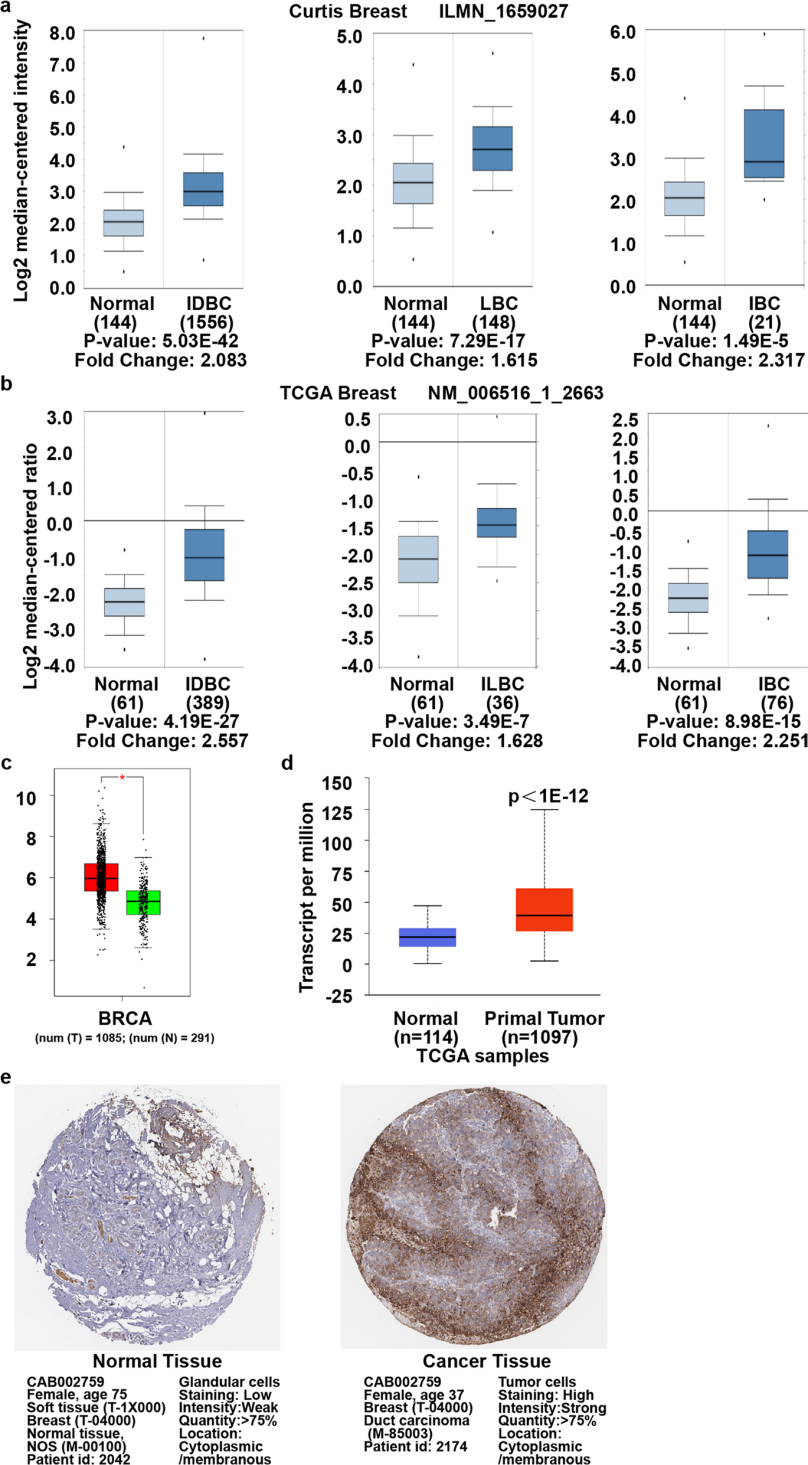
Transcription and Protein Levels are Upregulated in Breast Cancer versus Normal Sample. **a** and **b** Box plot of the mRNA expression from Curtis Breast dataset and TCGA Breast dataset (Oncomine database). IDBC: Invasive Ductal Breast Carcinoma, ILBC: Invasive Lobular Breast Carcinoma, IBC: Invasive Breast Carcinoma. **c** The transcription level in GEPIA database, *p* < 0.05. **d** The mRNA expression from UALCAN database, *p* < 1E− 12. **e** The protein expression from the Human Protein Atlas

GLUT1 mRNA level upregulated in 10 out of 11 datasets in breast cancer (Oncomine). The increases in invasive breast, ductal, and lobular carcinoma are similar in different assessments with Curtis Breast dataset [27] (Table 1 and Fig. 2a). In The Cancer Genome Atlas dataset (TCGA) (Table 1), it also increased in invasive breast (2.251 folds), invasive ductal breast (2.557 folds), and invasive lobular breast (1.628 folds) carcinoma compared to their normal counterparts (Fig. 2b). Similarly, augmented expression was observed in invasive breast carcinoma compared to normal tissue (1.643 folds) in Gluck Breast dataset [28] (Table 1). Zhao et al. [29] (Table 1) reported an increase in invasive ductal (2.8 folds) and in lobular (2.075 folds) breast carcinomas. The similar results were obtained with GEPIA (Fig. 2c) and UALCAN (Fig. 2d) web tool. In addition, GLUT1 protein expression upregulated significantly in breast cancer tissues (Fig. 2e).

**Table 1:** Upregulation of GLUT1 between Different Types of Breast Cancer versus Normal Tissues (Oncomine)

	Type of Breast Cancer versus Normal Tissue	Fold Change	*p* Value	*t* Test	Source / Reference
Curtis Breast	Invasive Breast Carcinoma	2.317	1.49E-05	5.207	[27]
	Invasive Ductal and Invasive Lobular Breast Carcinoma	1.634	4.26E-14	8.040	[27]
	Invasive Ductal Breast Carcinoma	2.083	5.03E-42	17.681	[27]
	Invasive Lobular Breast Carcinoma	1.615	7.29E-17	8.779	[27]
	Breast Carcinoma	1.915	0.001	3.737	[27]
	Tubular Breast Carcinoma	1.614	1.80E-12	7.586	[27]
	Ductal Breast Carcinoma in Situ	2.055	0.004	3.329	[27]
	Mucinous Breast Carcinoma	2.100	6.44E-13	8.635	[27]
	Medullary Breast Carcinoma	2.728	4.82E-10	8.059	[27]
TCGA Breast	Invasive Breast Carcinoma	2.251	8.98E-15	8.629	TCGA
	Invasive Ductal Breast Carcinoma	2.557	4.19E-27	13.974	TCGA
	Invasive Lobular Breast Carcinoma	1.628	3.49E-07	5.359	TCGA
Gluck Breast	Invasive Breast Carcinoma	1.643	8.18E-12	9.369	[28]
Zhao Breast	Invasive Ductal Breast Carcinoma	2.800	1.03E-11	9.276	[29]
	Lobular Breast Carcinoma	2.075	6.62E-06	5.631	[29]

### Relation of GLUT1 mRNA and pathological characteristics

Using the UALCAN web tool (Table 2), we found that GLUT1 significantly upregulated in breast cancer independence of race (Additional file 1: Fig. S1 a), age (Additional file 1: Fig. S1 b), nodal metastasis status (Additional file 1: Fig. S1 d) and menopause status (Additional file 1: Fig. S1 g). The expression also significantly increased in different stages except stage 4 (Additional file 1: Fig. S1 c), probably because of the small sample size of stage 4. GLUT1 increased in almost all major subclasses (with TNBC types) except TNBC-LAR and TNBC-MSL (Additional file 1: Fig. S1 e). Apart from metaplastic, it increased in all histology subtypes (Additional file 1: Fig. S1 f).

**Table 2:** Relationship between GLUT1 mRNA Expression and Clinicopathological Characteristics in Breast Cancer (TCGA data)

Clinicopathologic Characteristics	mRNA Expression	# of Sample(n)	*p*-Value
Sample types			
Normal	↓	114	
Primary tumor	↑	1097	<1E-12
Patient’s race			
Normal	↓	114	
Caucasian	↑	748	1.62E-12
African-American	↑	179	2.44E-10
Asian	↑	61	4.02E-10
Patient’s age			
Normal	↓	114	
21–40 Yrs.	↑	97	1.36E-10
41–60 Yrs.	↑	505	<1E-12
61–80 Yrs.	↑	431	1.11E-16
81–100 Yrs.	↑	54	1.63E-04
Individual cancer stages			
Normal	↓	114	
Stage 1	↑	183	2.61E-14
Stage 2	↑	615	1.62E-12
Stage 3	↑	247	1.62E-12
Stage 4	↑	20	1.10E-01
Major subclasses (with TNBC types)			
Normal	↓	114	
Luminal	↑	556	1.62E-12
HER2Pos	↑	37	1.94E-03
TNBC-BL1	↑	13	2.97E-04
TNBC-BL2	↑	11	3.24E-03
TNBC-IM	↑	20	3.60E-03
TNBC-LAR	↑	8	8.89E-02
TNBC-MSL	↑	8	8.30E-02
TNBC-M	↑	29	8.28E-05
TNBC-UNS	↑	27	1.75E-04
Tumor histology			
Normal	↓	114	
IDC	↑	784	1.62E-12
ILC	↑	203	1.68E-12
Mixed	↑	29	2.30E-06
Other	↑	45	1.62E-04
Mucinous	↑	17	2.90E-03
Metaplastic	↑	9	2.67E-01
INOS	↑	1	N/A
Medullary	↑	6	2.90E-02
Nodal Metastasis status			
Normal	↓	114	
N0	↑	516	1.62E-12
N1	↑	362	1.62E-12
N2	↑	120	3.98E-08
N3	↑	77	3.53E-09
Menopause status			
Normal	↓	114	
Pre-Menopause	↑	230	1.11E-16
Peri-Menopause	↑	37	3.00E-03
Post-Menopause	↑	700	1.62E-12

### GLUT1 associated with poor prognosis and metastasis

**Fig. 3:**
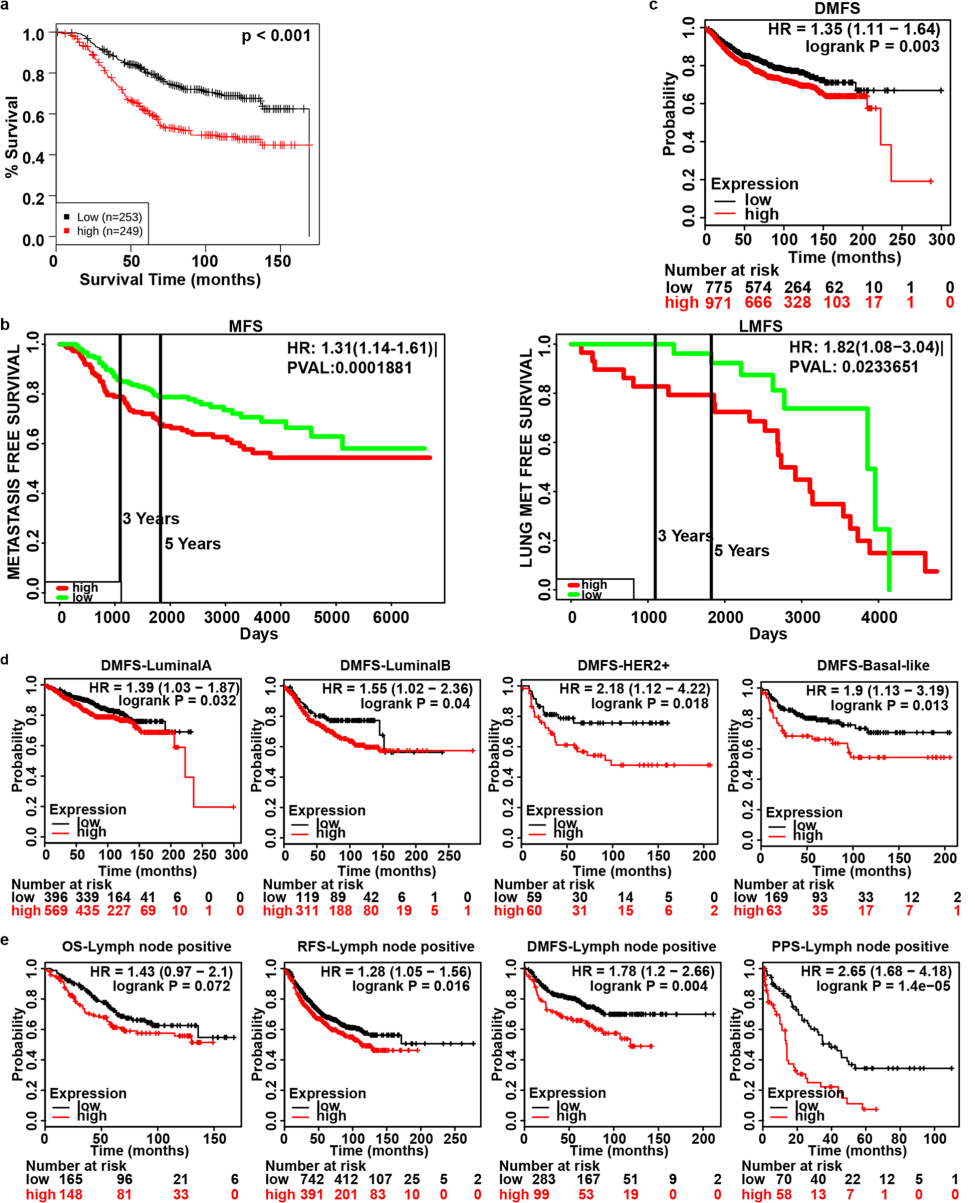
GLUT1 mRNA Level and Survival in Breast Cancer. **a** Overall Survival (OS) from GENT2 database (*p*<0.001). **b** Metastasis Free Survival (MFS, *p*<0.001) and Lung Metastasis Free Survival (LMFS, *p* = 0.023) from PROGgeneV2 database. **c** and **d** Distant Metastasis Free Survival (DMFS) (*p* = 0.003) in all patients and with its subclasses respectively from Kaplan-Meier Plotter. Luminal A: *p* = 0.032, Luminal B: *p* = 0.04, HER2+: *p* = 0.018, Basal-like: *p* = 0.013. **e** OS (*p* = 0.072), Recurrence-Free Survival (RFS, *p* = 0.016), DMFS (*p* = 0.004) and Post-Progression Survival (PPS, *p* = 1.4e-05) in lymph node positive breast cancer from Kaplan-Meier Plotter

GLUT1 expression was negatively correlated with OS (Fig. 3a) (GENT database), metastasis free survival and lung metastasis free survival (Fig. 3b, Table 3) (PROGgeneV2 database) of patients. High mRNA expression of GLUT1 was predicted to decrease DMFS in all patients (Fig. 3c) with different subclasses (Fig. 3d). It was also negatively correlated with OS, RFS, DMFS and PPS in lymph node positive breast cancer (Fig. 3e). Thus, the Kaplan-Meier curve and log rank test analyses revealed that the increase was significantly associated with recurrence, distant metastasis and poor prognosis.

**Table 3:** Survival Analysis of GLUT1 mRNA Expression in Breast Cancer (PROGgeneV2)

Survival analysis	Dataset	# of Sample (n)	*p* Value	HR	HR [95% CI]
Overall Survival	GSE21653	247	0.000815	1.79	[1.27–2.51]
	GSE3494_U133A	236	0.004264	2.05	[1.25–3.34]
	GSE42568	104	0.022628	1.37	[1.04–1.79]
	GSE22133 − GPL5345	349	0.027383	1.29	[1.03–1.61]
Relapse Free Survival	GSE22219	215	0.000004	2.44	[1.67–3.56]
	GSE17705	298	0.005359	2.77	[1.35–5.66]
	GSE4922_U133A	249	0.041596	1.59	[1.02–2.49]
	GSE2034	286	0.041810	1.41	[1.01–1.96]
Metastasis Free Survival	NKI	295	0.000188	1.31	[1.14–1.51]
Lung Met Free Survival	GSE5327	58	0.023365	1.82	[1.08–3.04]

### Mutations, methylation and copy number alteration analysis

**Fig. 4:**
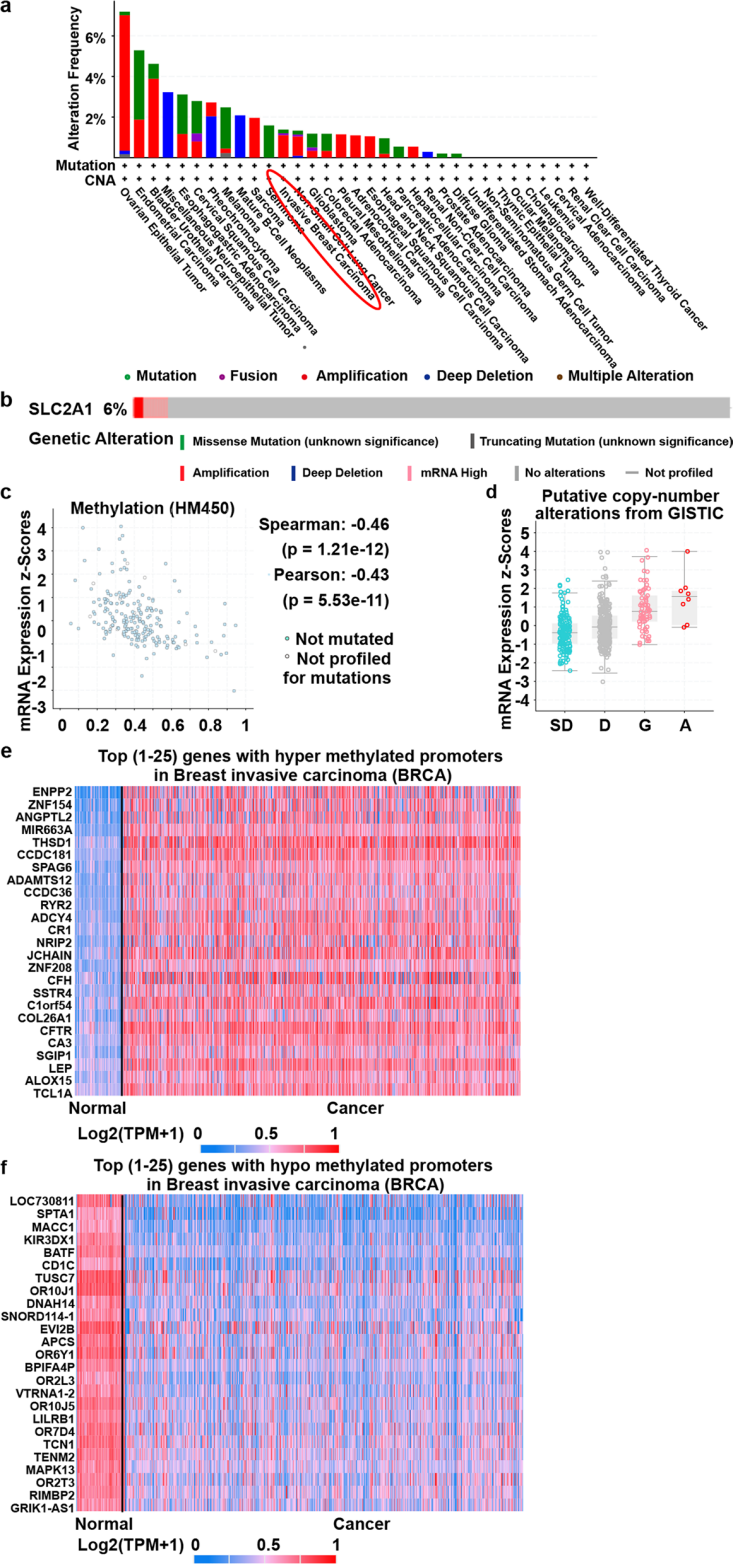
Relation of GLUT1 to Mutations, Methylation and Copy Number Alterations in Breast Cancer. **a** Mutations in 33 types of cancer. Altered in 195 (2%) of 10,967 sequenced patiens (10,967 total) from TCGA PanCancer Atlas Studies (cBioPortal web tool). **b** Mutations in breast cancer. Altered in 65 (6%) of 1108 sequenced patiens (1108 total) from TCGA, Firehose Legacy (cBioPortal web tool). **c** Methylation level using spearman (*r* = − 0.46, *p* = 1.21e− 12) and pearson (*r* = − 0.43, *p* = 5.53e− 11) from TCGA, Firehose Legacy (cBioPortal web tool). **d** Copy number alterations from TCGA, Firehose Legacy (cBioPortal web tool). SD: Shallow Deletion, D: Diploid, G: Gain, A: Amplification. **e** and **f** Heatmap of top [1–25] genes with hyper and hypo methylated promoters, respectively (UALCAN database)

We further analyzed GLUT1 mutation, methylation and copy number alterations. Mutations were altered in about 2% of sequenced patients in 33 types of cancer (Fig. 4a) and 6% of sequenced patients in breast cancer (Fig. 4b) (cBioPortal web tools). Methylation was negatively correlated with mRNA expression with spearman and pearson analyses (Fig. 4c). In the copy number variation analysis (CNAs), amplification and gain were predominantly correlated with GLUT1 expression (Fig. 4d). These results suggest that the overexpression of GLUT1 could partly result from alterations in methylation and CNAs. Heatmaps of top [1–25] genes with hyper (Fig. 4e) and hypo (Fig. 4f) methylated promoters were also examined with UALCAN web tools.

### Relation of GLUT1 and JUN and their expression in human breast cancer tissues

We investigated the transcription factors in GeneCard and PROMO databases and found that transcription factor c-Jun encoded by gene JUN can bind to the GLUT1 promoter. Prediction of the binding site sequences suggest that the binding may regulate the GLUT1 gene expression (Table 4).

**Table 4:** The Predicted Binding Site Sequences of Transcription Factor c-Jun and GLUT1 Promoter (JASPAR)

Model ID	Model name	Score	Relative score	Start	End	Strand	predicted site sequence
MA0488.1	JUN	6.136	0.854547759	258	270	-1	AGGGGGAGGTCAT
MA0488.1	JUN	4.846	0.840498242	419	431	1	ACAATGGGGTCAC
MA0488.1	JUN	4.976	0.841914085	422	434	-1	CATGTGACCCCAT
MA0488.1	JUN	2.451	0.814414063	909	921	1	ATGGTGAGGTCGG
MA0488.1	JUN	3.442	0.825207141	947	959	1	AAGCTGAGCAAAT
MA0488.1	JUN	3.089	0.821362584	1004	1016	1	AAACTGGTGCCAT
MA0488.1	JUN	4.920	0.841304184	1539	1551	1	AAGGTGAAGTCAG
MA0488.1	JUN	1.133	0.800059597	1736	1748	1	TTCATGAGCTTAT
MA0488.1	JUN	2.281	0.812562577	1739	1751	-1	CAGATAAGCTCAT
MA0488.1	JUN	2.526	0.815230896	1914	1926	1	AAAATGGTGAAAC
MA0488.1	JUN	1.854	0.807912078	2226	2238	1	CCTATCATGTCAG
MA0488.1	JUN	3.561	0.826503182	2541	2553	-1	CAAGTGAGTTAAT

**Fig. 5:**
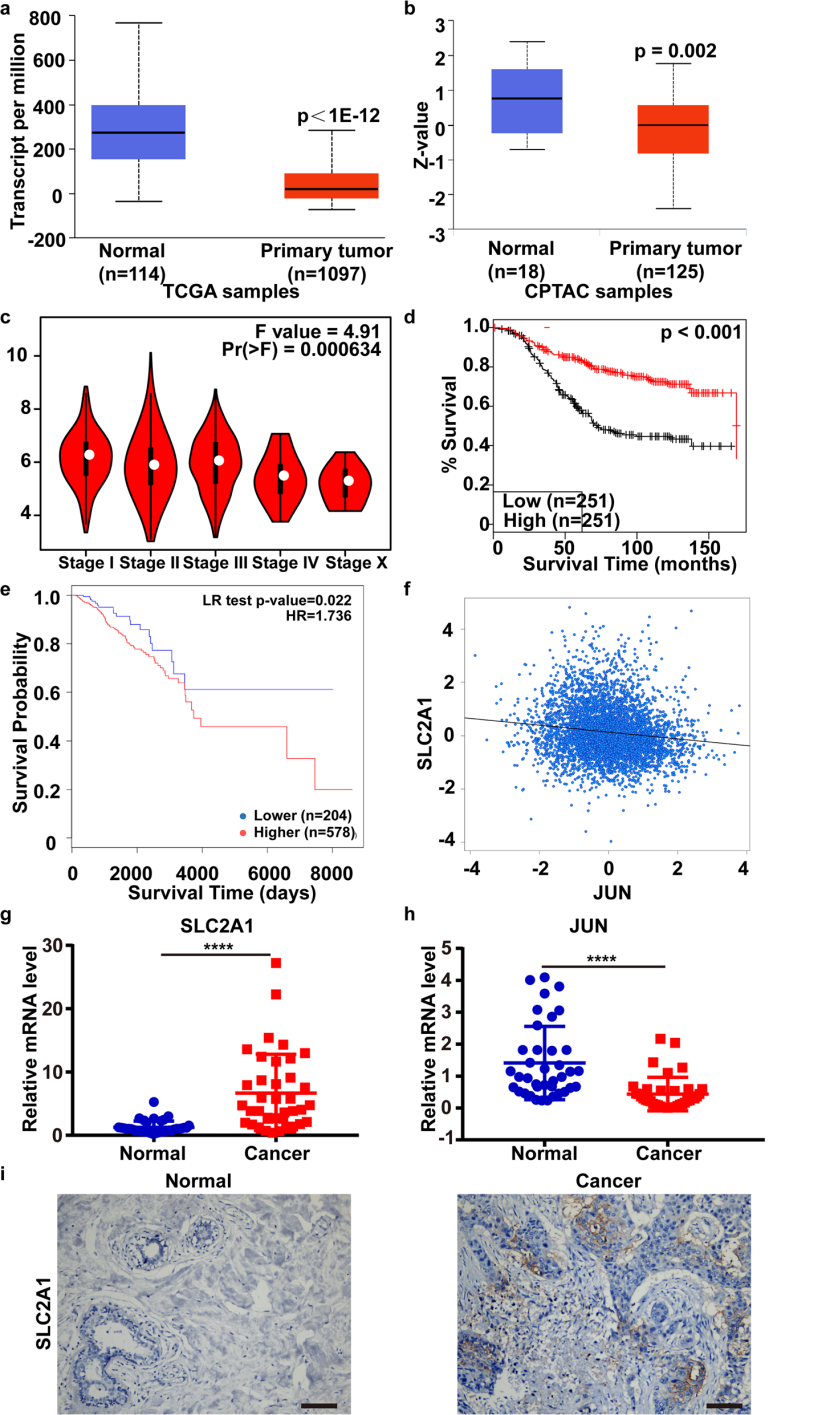
Relation of GLUT1 and JUN and their expression in human breast cancer tissues. **a** and **b** The mRNA expression and protein phosphorylation at T239S243 of JUN (UALCAN database). **c** Tumor stage, *p* = 0.000634 (GEPIA database). **d** Overall survival, *p* < 0.001 (GENT2 database). **e** Relationship between methylation (CpG island at 5’UTR and 1stExon of JUN) and overall survival (MethSurv web tool). **f** The correlation of JUN and GLUT1, *p* = 0.0001, *r* = − 0.13 (bc-GenExMiner web tool). **g** and **h** The mRNA level of GLUT1 and JUN in 38 paired human breast cancer samples by qRT-PCR. *p < 0.05, **p < 0.01, ***p < 0.001 ****p < 0.0001. Error bars, mean ± SD. **i** IHC results of GLUT1. Scale bars, 50 μm

We analyzed the expression and prognostic value of JUN. The mRNA expression (*p* < 1E− 12) and protein phosphorylation level at T239S243 (*P* = 0.002) of JUN significantly decreased in breast cancer (UALCAN database) (Fig. 5a, b). JUN expression varied with tumor stages (Fig. 5c), and was positively correlated with OS (Fig. 5d). In addition, the decreased methylation level was associated with increased overall survival (*p* = 0.022, HR = 1.736), indicating that the low expression of JUN might be partly due to methylation (Fig. 5e). GLUT1 expression was negatively correlated with JUN (*p* = 0.0001, *r* = − 0.13) in the bc-GenExMiner web tool (Fig. 5f).

Furthermore, GLUT1 was upregulated in 38 paired human breast cancer tissues while JUN was downregulated by qRT-PCR (Fig. 5g, h). Through IHC, we verified that the protein level of GLUT1 was significantly increased in tumor samples (Fig. 5i).

### Pathway analysis of alterations in GLUT1 and JUN and their co-expressed genes

We examined genes that co-expressed with GLUT1 and JUN in breast cancer in Adorno cell line dataset [30] (Additional file 2: Fig. S2 a, Additional file 2: Fig. S2 b), and found that the transcription level of GLUT1 and JUN were closely corelated and might contribute to certain signaling pathways. Hence, we performed GO and KEGG analysis of genes associated with GLUT1 and JUN.

**Fig. 6:**
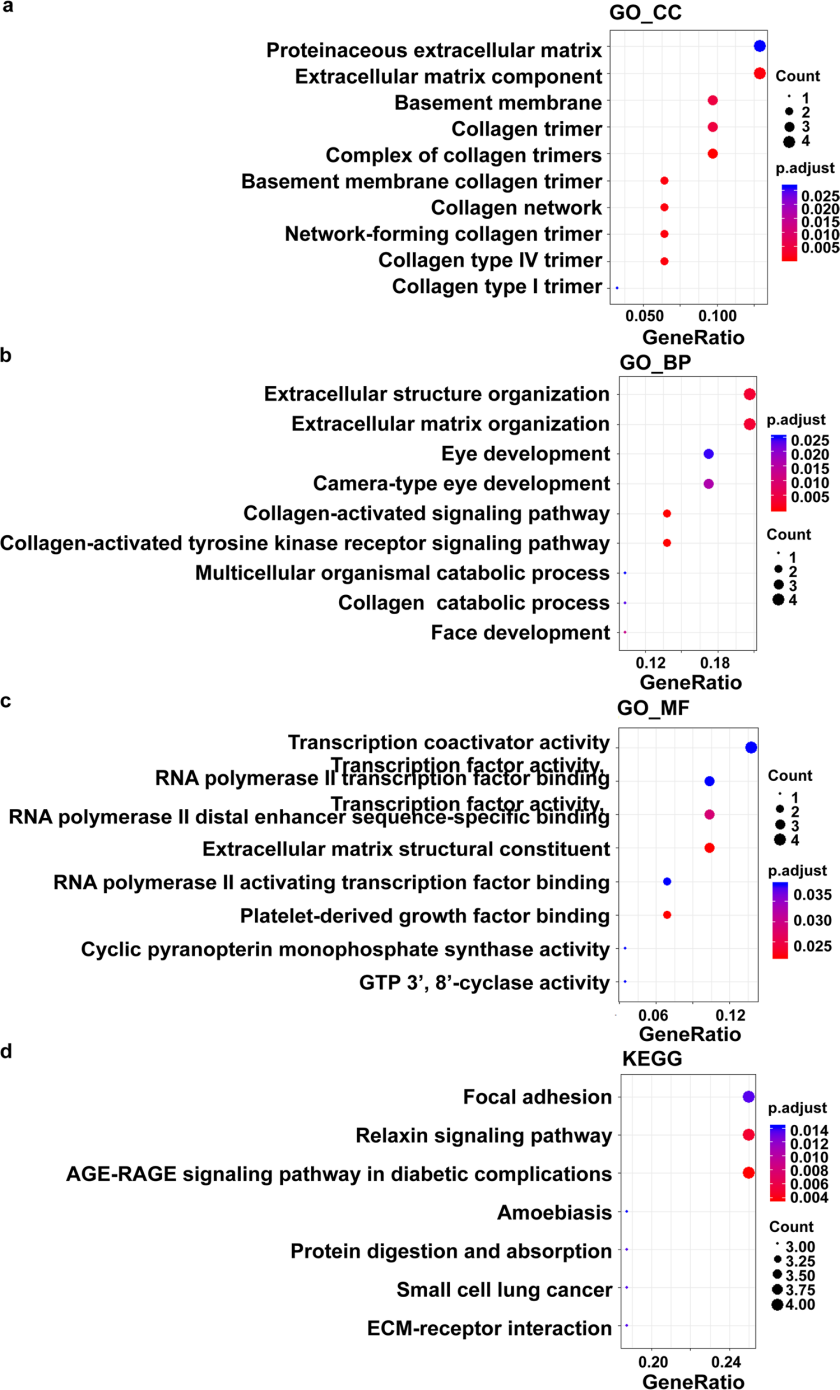
GO and KEGG Analysis (Correlation with GLUT1 and JUN by R/Bioconductor). **a** Cellular components. **b** Biological processes. **c** Molecular functions. **d** KEGG pathway

In GO analysis, both GLUT1 and JUN regulated three aspects, i.e. cellular components [proteinaceous extracellular matrix, extracellular matrix component, basement membrane and collagen trimer (Fig. 6a)], biological processes [Extracellular structure organization, extracellular matrix organization and collagen-activated signaling pathway (Fig. 6b)], and molecular functions [Transcription coactivator activity, transcription factor activity (Fig. 6c)].

In KEGG analysis, GLUT1 and JUN are associated with seven breast cancer related pathways. Among them, focal adhesion and ECM-receptor interaction were involved in tumorigenesis and metastasis (Fig. 6d, Additional file 3: Fig. S3).

## Discussion

GLUT1 plays a role in tumorigenesis, metastasis and prognosis in many cancers [7, 10, 11, 31]. The present study is the first to use bioinformatics and laboratory experiments to demonstrate that the increased mRNA expression of GLUT1 was also associated with poor prognosis and metastasis in breast cancer, which may be regulated via JUN by binding to GLUT1 promoter.

In oral squamous cell carcinoma, miR-378a-3p represses GLUT1 expression by targeting its 3’UTR and inhibits cell migration and invasion both in vitro and in vivo [7]. Genetic or pharmacological inhibition of GLUT1 with BAY-876 impairs the growth of a subset of TNBC cells displaying high glycolytic and lower oxidative phosphorylation (OXPHOS) rates [32]. In our study, GLUT1 mRNA expression was much higher in breast cancer and significantly associated with the clinicopathological characteristics (race, age, metastasis and menopause status). Moreover, it was negatively correlated with OS, metastasis free survival and lung metastasis free survival. In addition, it was significantly associated with poor OS, RFS, DMFS and PPS in breast cancer patients with positive lymph node by Kaplan-Meier curve analysis. Thus, inhibition of GLUT1 might suppress breast cancer proliferation and metastasis.

Transcription factor c-Jun is closely associated with metastasis, prognosis and acts as both suppressor and oncogene. Increased expression (by inhibition of ATF2 via RNA interference) leads melanoma to undergo cell apoptosis and inhibits growth and metastasis [33]. Moreover, it binds to the promoter region of human miR-374a and suppress miR-374a in A549 and pc-9 cells, leading to inhibition of cell growth, migration, invasion and metastasis [34]. However, c-Jun may promote tumorigenesis and metastasis. For example, it binds and activates human FOXK1 (Forkhead box (FOX) K1) gene promoter, enhancing proliferation and metastasis in gastric cancer [35]. Transcription factors (NFATc, ATF2, and c-Jun) upregulated in lung cancer aggressive cells, increasing angiopoietin-like protein 2 (ANGPTL2) expression, and cell motility and invasion [36]. In gastric cancer, SIRT1 suppresses migration and invasion by downregulating ARHGAP5 through inhibiting c-Jun [37]. Moreover, c-Jun is closely related to glucose metabolism. TGF-β1b/c-Jun targets G6PC3 of FoxO signaling to promote gluconeogenesis and maintain a high glucose level [38]. c-Jun also binds to and activates the β1,3-N-acetyl-glucosaminyltrans-ferase8 (β3Gn-T8) promoter and thus upregulates the β3Gn-T8 expression, leading to malignancy in gastric cancer [39]. However, mechanisms of controlling the interplay between GLUT1 and c-Jun remain to be elucidated. In our report, the mRNA expression and protein phosphorylation at T239S243 of JUN were significantly downregulated in breast cancer tissues. The low expression of JUN might be partly due to methylation. In addition, the JUN expression and the methylation were correlated with survival. The expressions of GLUT1 and JUN were negatively correlated in the bc-GenExMiner web. Thus c-Jun might bind to GLUT1 promoter at several sites to suppress its expression.

In GO and KEGG analysis [40], GLUT1 and JUN regulated proteinaceous extracellular matrix, extracellular structure organization and transcription coactivator activity and modulate focal adhesion and ECM-receptor interaction signaling pathways, which associated with metastasis.

The mRNA and protein levels of GLUT1 are highly expressed in breast cancer, while c-Jun is low expression. The results of correlation analysis in this study show that there is a negative correlation between the two. The enrichment analysis of the related genes also demonstrated that GLUT1 and c-jun were associated with the signal pathways such as modulated focal adhesion and ECM-receptor interaction signaling pathways, which were closely related to the occurrence and development of breast cancer. Therefore, the interaction between GLUT1 and c-jun regulating glucose metabolism may be a potential mechanism for the growth and metastasis of breast cancer.

## Conclusions

High expression of GLUT1 is significantly associated with poor prognosis and promotes glucose metabolism and tumor metastasis. The GLUT1 effect may be regulated by hypomethylation and JUN expression. Thus, GLUT1 could be a novel target for breast cancer treatment.

## Supplementary Information

**Additional file 1 **: **Fig. S1**. Relationship between GLUT1 Transcription Level and Clinicopathological Characteristics in Breast Cancer (UALCAN). a. Race. b. Age. c. Cancer stage. d. Metastasis status (number of axillary lymph nodes involved), N0: 0, N1: 1 to 3, N2: 4 to 9, N3: 10 or more. e. Major subclass (with TNBC types), TNBC-BL1: TNBC Basal-like 1, TNBC-BL2: TNBC Basal-like 2, TNBC-IM: TNBC, Immunomodulatory, TNBC-LAR: TNBC luminal androgen receptor, TNBC-MSL: TNBC mesenchymal stem-like, TNBC-M: TNBC Mesenchymal, TNBC-UNS: TNBC unspecified. f. Histology, IDC: Infiltrating Ductal Carcinoma, ILC: Infiltrating Lobular Carcinoma, Mixed: Mixed histology, Mucinous: Mucinous Carcinoma, Metaplastic: Metaplastic Carcinoma, INOS: Infiltrating Carcinoma NOS, Medullary: Medullary Carcinoma. g. Menopause status.

**Additional file 2 **: **Fig. S2**. Heatmap of Genes Co-expressed with GLUT1 and JUN in Breast Cancer (Oncomine database). a. and b. Genes co-expressed with GLUT1 and JUN respectively in Adorno cell line.

**Additional file 3 **: **Fig. S3**. Signaling Pathways Regulated by Genes Correlated with GLUT1 and JUN in Breast Cancer. a. Focal adhesion. b. ECM-receptor interaction.

## Data Availability

The datasets generated during the current study are available in the official website of each database. All web links and accessions which can allow access to public data were listed in detail on materials and methods section. The official website of each database is as follows: Oncomine database (https://www.oncomine.org/resource/login.html-The website has been shut down from 17.Jan 2022). This data will be available from the corresponding author on reasonable request. GEPIA (http://gepia.cancer-pku.cn/index.html). GENT database (http://gent2.appex.kr/gent2/). UALCAN (http://ualcan.path.uab.edu/). cBioPortal (https://www.cbioportal.org/). The Human Protein Atlas (https://www.proteinatlas.org/). Kaplan-Meier Plotter (http://kmplot.com/analysis/index.php?p=background). PROGgeneV2 database (http://www.progtools.net/gene/). MethSurv web tool (https://biit.cs.ut.ee/methsurv). This data will be available from the corresponding author on reasonable request. bc-GenExMiner Web Tool (http://bcgenex.ico.unicancer.fr).

## References

[1] Sung H, Ferlay J, Siegel RL, Laversanne M, Soerjomataram I, Jemal A (2021). Global Cancer statistics 2020: GLOBOCAN estimates of incidence and mortality worldwide for 36 cancers in 185 countries. CA Cancer J Clin.

[2] Anderson RL, Balasas T, Callaghan J, Coombes RC, Evans J, Hall JA (2019). A framework for the development of effective anti-metastatic agents. Nat Rev Clin Oncol.

[3] Spano D, Heck C, De Antonellis P, Christofori G, Zollo M (2012). Molecular networks that regulate cancer metastasis. Semin Cancer Biol.

[4] Warburg O, Wind F, Negelein E (1927). The metabolism of tumors in the body. J Gen Physiol.

[5] Cantor JR, Sabatini DM (2012). Cancer cell metabolism: one hallmark, many faces. Cancer Discov.

[6] Vander Heiden MG, Cantley LC, Thompson CB (2009). Understanding the Warburg effect: the metabolic requirements of cell proliferation. Science.

[7] Wang Y, Zhang X, Wang Z, Hu Q, Wu J, Li Y (2018). LncRNA-p23154 promotes the invasion-metastasis potential of oral squamous cell carcinoma by regulating Glut1-mediated glycolysis. Cancer Lett.

[8] Counihan JL, Grossman EA, Nomura DK (2018). Cancer metabolism: current understanding and therapies. Chem Rev.

[9] Wilson WR, Hay MP (2011). Targeting hypoxia in cancer therapy. Nat Rev Cancer.

[10] Goos JA, de Cuba EM, Coupe VM, Diosdado B, Delis-Van Diemen PM, Karga C (2016). Glucose transporter 1 (SLC2A1) and vascular endothelial growth factor a (VEGFA) predict survival after resection of colorectal Cancer liver metastasis. Ann Surg.

[11] Papageorgis P, Cheng K, Ozturk S, Gong Y, Lambert AW, Abdolmaleky HM (2011). Smad4 inactivation promotes malignancy and drug resistance of colon cancer. Cancer Res.

[12] Li W, Wei Z, Liu Y, Li H, Ren R, Tang Y (2010). Increased 18F-FDG uptake and expression of Glut1 in the EMT transformed breast cancer cells induced by TGF-beta. Neoplasma.

[13] Jiang P, Du W, Wang X, Mancuso A, Gao X, Wu M (2011). p53 regulates biosynthesis through direct inactivation of glucose-6-phosphate dehydrogenase. Nat Cell Biol.

[14] Boidot R, Vegran F, Meulle A, Le Breton A, Dessy C, Sonveaux P (2012). Regulation of monocarboxylate transporter MCT1 expression by p53 mediates inward and outward lactate fluxes in tumors. Cancer Res.

[15] Chen XX, Yin Y, Cheng JW, Huang A, Hu B, Zhang X (2018). BAP1 acts as a tumor suppressor in intrahepatic cholangiocarcinoma by modulating the ERK1/2 and JNK/c-Jun pathways. Cell Death Dis.

[16] Zhu P, Lu J, Zhi X, Zhou Y, Wang X, Wang C (2021). tRNA-derived fragment tRFLys-CTT-010 promotes triple-negative breast cancer progression by regulating glucose metabolism via G6PC. Carcinogenesis.

[17] Rhodes DR, Yu J, Shanker K, Deshpande N, Varambally R, Ghosh D (2004). ONCOMINE: a cancer microarray database and integrated data-mining platform. Neoplasia.

[18] Tang Z, Li C, Kang B, Gao G, Li C, Zhang Z (2017). GEPIA: a web server for cancer and normal gene expression profiling and interactive analyses. Nucleic Acids Res.

[19] Park SJ, Yoon BH, Kim SK, Kim SY (2019). GENT2: an updated gene expression database for normal and tumor tissues. BMC Med Genet.

[20] Chandrashekar DS, Bashel B, Balasubramanya SAH, Creighton CJ, Ponce-Rodriguez I, Chakravarthi B (2017). UALCAN: a portal for facilitating tumor subgroup gene expression and survival analyses. Neoplasia.

[21] Cerami E, Gao J, Dogrusoz U, Gross BE, Sumer SO, Aksoy BA (2012). The cBio cancer genomics portal: an open platform for exploring multidimensional cancer genomics data. Cancer Discov.

[22] Uhlen M, Fagerberg L, Hallstrom BM, Lindskog C, Oksvold P, Mardinoglu A (2015). Proteomics. Tissue-based map of the human proteome. Science.

[23] Gyorffy B, Lanczky A, Eklund AC, Denkert C, Budczies J, Li Q (2010). An online survival analysis tool to rapidly assess the effect of 22,277 genes on breast cancer prognosis using microarray data of 1,809 patients. Breast Cancer Res Treat.

[24] Goswami CP, Nakshatri H (2014). PROGgeneV2: enhancements on the existing database. BMC Cancer.

[25] Modhukur V, Iljasenko T, Metsalu T, Lokk K, Laisk-Podar T, Vilo J (2018). MethSurv: a web tool to perform multivariable survival analysis using DNA methylation data. Epigenomics.

[26] Jezequel P, Campone M, Gouraud W, Guerin-Charbonnel C, Leux C, Ricolleau G (2012). Bc-GenExMiner: an easy-to-use online platform for gene prognostic analyses in breast cancer. Breast Cancer Res Treat.

[27] Curtis C, Shah SP, Chin SF, Turashvili G, Rueda OM, Dunning MJ (2012). The genomic and transcriptomic architecture of 2,000 breast tumours reveals novel subgroups. Nature.

[28] Gluck S, Ross JS, Royce M, McKenna EF, Perou CM, Avisar E (2012). TP53 genomics predict higher clinical and pathologic tumor response in operable early-stage breast cancer treated with docetaxel-capecitabine +/− trastuzumab. Breast Cancer Res Treat.

[29] Zhao H, Langerod A, Ji Y, Nowels KW, Nesland JM, Tibshirani R (2004). Different gene expression patterns in invasive lobular and ductal carcinomas of the breast. Mol Biol Cell.

[30] Adorno M, Cordenonsi M, Montagner M, Dupont S, Wong C, Hann B (2009). A mutant-p53/Smad complex opposes p63 to empower TGFbeta-induced metastasis. Cell.

[31] Qin Q, Yang B, Liu J, Song E, Song Y (2021). Polychlorinated biphenyl quinone exposure promotes breast cancer aerobic glycolysis: An in vitro and in vivo examination. J Hazard Mater.

[32] Wu Q, Ba-Alawi W, Deblois G, Cruickshank J, Duan S, Lima-Fernandes E (2020). GLUT1 inhibition blocks growth of RB1-positive triple negative breast cancer. Nat Commun.

[33] Bhoumik A, Huang TG, Ivanov V, Gangi L, Qiao RF, Woo SL (2002). An ATF2-derived peptide sensitizes melanomas to apoptosis and inhibits their growth and metastasis. J Clin Invest.

[34] Zhao M, Xu P, Liu Z, Zhen Y, Chen Y, Liu Y (2018). Dual roles of miR-374a by modulated c-Jun respectively targets CCND1-inducing PI3K/AKT signal and PTEN-suppressing Wnt/beta-catenin signaling in non-small-cell lung cancer. Cell Death Dis.

[35] Peng Y, Zhang P, Huang X, Yan Q, Wu M, Xie R (2016). Direct regulation of FOXK1 by C-Jun promotes proliferation, invasion and metastasis in gastric cancer cells. Cell Death Dis.

[36] Endo M, Nakano M, Kadomatsu T, Fukuhara S, Kuroda H, Mikami S (2012). Tumor cell-derived angiopoietin-like protein ANGPTL2 is a critical driver of metastasis. Cancer Res.

[37] Dong G, Wang B, An Y, Li J, Wang X, Jia J (2018). SIRT1 suppresses the migration and invasion of gastric cancer by regulating ARHGAP5 expression. Cell Death Dis.

[38] Zhang CY, Yin HM, Wang H, Su D, Xia Y, Yan LF (2018). Transforming growth factor-beta1 regulates the nascent hematopoietic stem cell niche by promoting gluconeogenesis. Leukemia.

[39] Jiang Z, Liu Z, Zou S, Ni J, Shen L, Zhou Y (2016). Transcription factor c-Jun regulates beta3Gn-T8 expression in gastric cancer cell line SGC-7901. Oncol Rep.

[40] Kanehisa M, Furumichi M, Sato Y, Ishiguro-Watanabe M, Tanabe M (2021). KEGG: integrating viruses and cellular organisms. Nucleic Acids Res.

