# Supplementary figures and images for: Transcription factor c-Jun modulates GLUT1 in glycolysis and breast cancer metastasis
Source: BMC Cancer. 2022 Dec 7;22:1283. doi: 10.1186/s12885-022-10393-x (PMC9730598; doi:10.1186/s12885-022-10393-x)

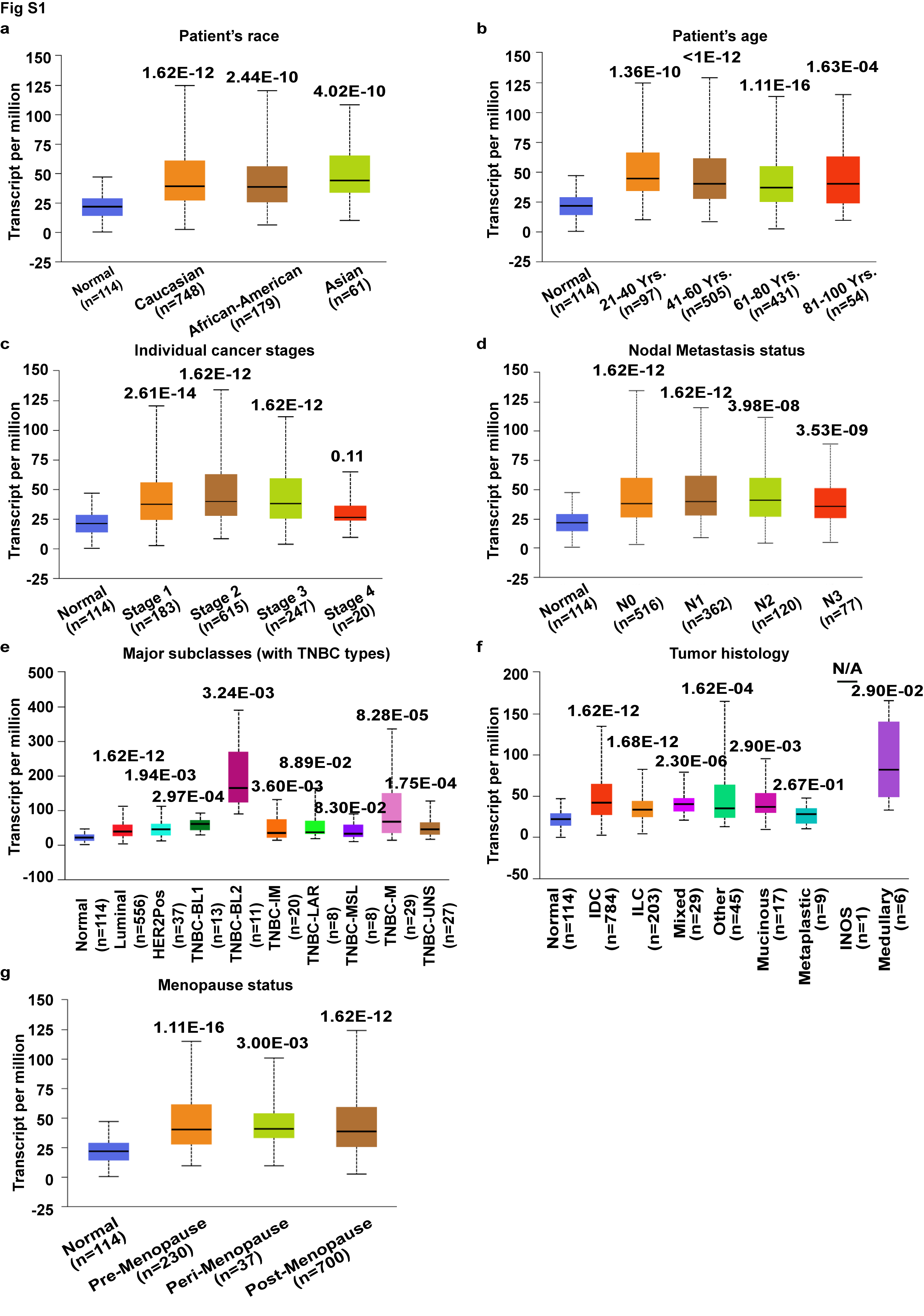

Supplement: Supplementary file 1 — Additional file 1 : Fig. S1. Relationship between GLUT1 Transcription Level and Clinicopathological Characteristics in Breast Cancer (UALCAN). a. Race. b. Age. c. Cancer stage. d. Metastasis status (number of axillary lymph nodes involved), N0: 0, N1: 1 to 3, N2: 4 to 9, N3: 10 or more. e. Major subclass (with TNBC types), TNBC-BL1: TNBC Basal-like 1, TNBC-BL2: TNBC Basal-like 2, TNBC-IM: TNBC, Immunomodulatory, TNBC-LAR: TNBC luminal androgen receptor, TNBC-MSL: TNBC mesenchymal stem-like, TNBC-M: TNBC Mesenchymal, TNBC-UNS: TNBC unspecified. f. Histology, IDC: Infiltrating Ductal Carcinoma, ILC: Infiltrating Lobular Carcinoma, Mixed: Mixed histology, Mucinous: Mucinous Carcinoma, Metaplastic: Metaplastic Carcinoma, INOS: Infiltrating Carcinoma NOS, Medullary: Medullary Carcinoma. g. Menopause status. [file 12885_2022_10393_MOESM1_ESM.tif]

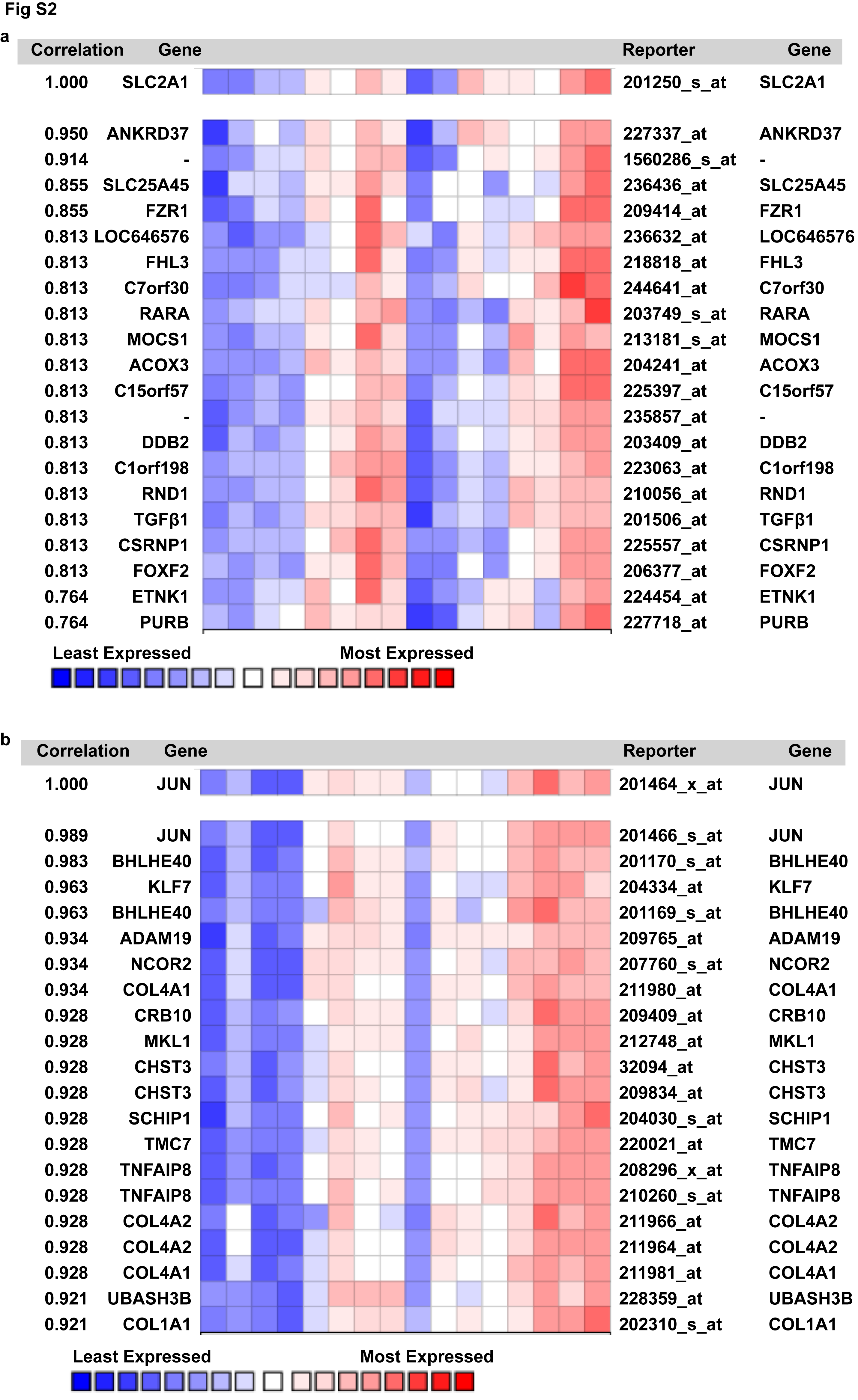

Supplement: Supplementary file 2 — Additional file 2 : Fig. S2. Heatmap of Genes Co-expressed with GLUT1 and JUN in Breast Cancer (Oncomine database). a. and b. Genes co-expressed with GLUT1 and JUN respectively in Adorno cell line. [file 12885_2022_10393_MOESM2_ESM.tif]

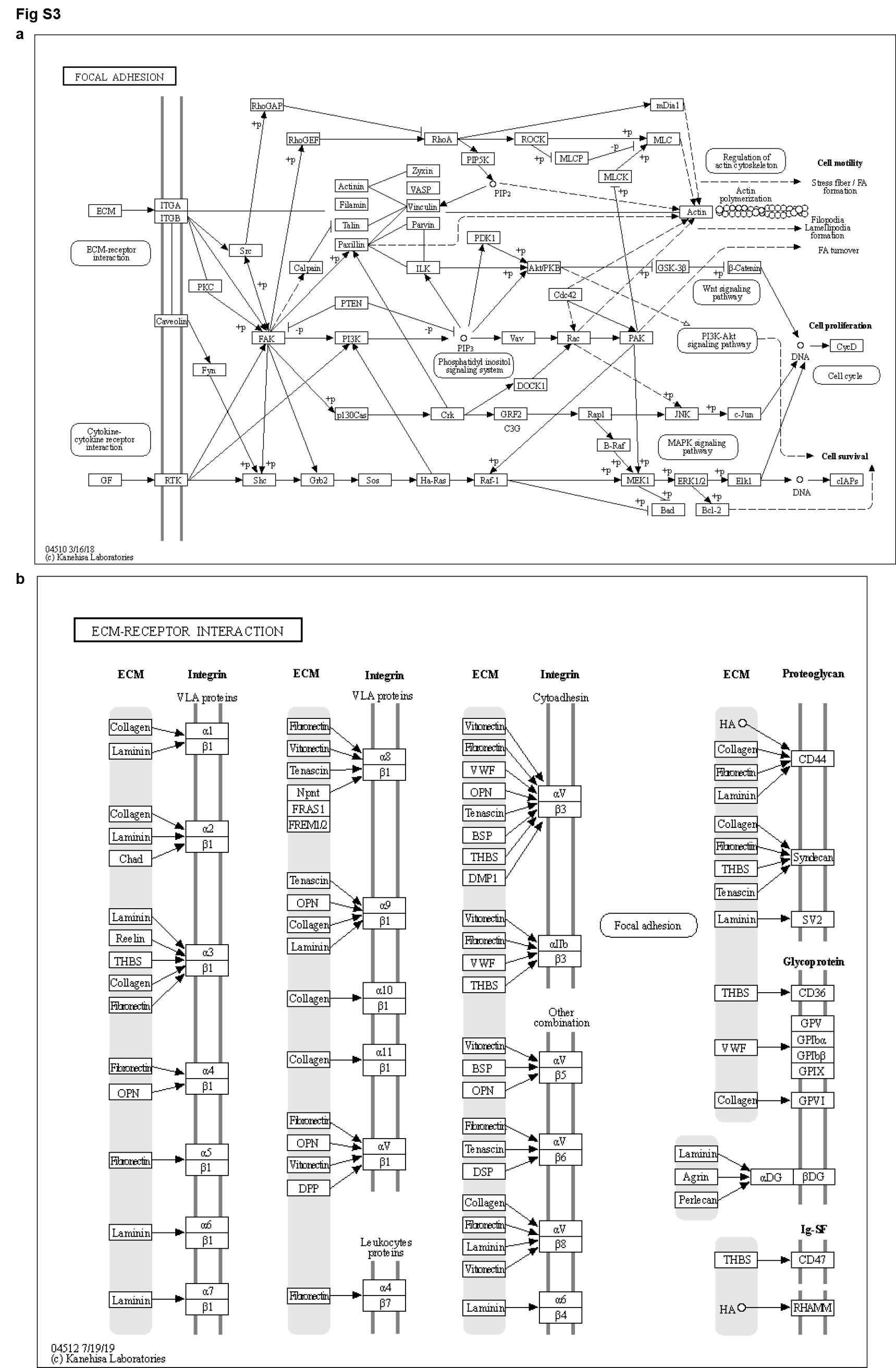

Supplement: Supplementary file 3 — Additional file 3 : Fig. S3. Signaling Pathways Regulated by Genes Correlated with GLUT1 and JUN in Breast Cancer. a. Focal adhesion. b. ECM-receptor interaction. [file 12885_2022_10393_MOESM3_ESM.tif]
